# Human Beta Defensins and Cancer: Contradictions and Common Ground

**DOI:** 10.3389/fonc.2019.00341

**Published:** 2019-05-03

**Authors:** Santosh K. Ghosh, Thomas S. McCormick, Aaron Weinberg

**Affiliations:** ^1^Biological Sciences, School of Dental Medicine, Case Western Reserve University, Cleveland, OH, United States; ^2^Dermatology, School of Medicine, Case Western Reserve University, Cleveland, OH, United States

**Keywords:** hBD-1, hBD-2, hBD-3, cancer, migration, proliferation

## Abstract

Human beta-defensins (hBDs, −1, 2, 3) are a family of epithelial cell derived antimicrobial peptides (AMPs) that protect mucosal membranes from microbial challenges. In addition to their antimicrobial activities, they possess other functions; e.g., cell activation, proliferation, regulation of cytokine/chemokine production, migration, differentiation, angiogenesis, and wound healing processes. It has also become apparent that defensin levels change with the development of neoplasia. However, inconsistent observations published by various laboratories make it difficult to reach a consensus as to the direction of the dysregulation and role the hBDs may play in various cancers. This is particularly evident in studies focusing on oral squamous cell carcinoma (OSCC). By segregating each hBD by cancer type, interrogating methodologies, and scrutinizing the subject cohorts used in the studies, we have endeavored to identify the “take home message” for each one of the three hBDs. We discovered that (1) consensus-driven findings indicate that hBD-1 and−2 are down- while hBD-3 is up-regulated in OSCC; (2) hBD dysregulation is cancer-type specific; (3) the inhibition/activation effect an hBD has on cancer cell lines is related to the direction of the hBD dysregulation (up or down) in the cancer from which the cell lines derive. Therefore, studies addressing hBD dysregulation in various cancers are not generalizable and comparisons should be avoided. Systematic delineation of the fate and role of the hBDs in a specific cancer type may lead to innovative ways to use defensins as prospective biomarkers for diagnostic/prognostic purposes and/or in novel therapeutic modalities.

## Introduction

The discovery of human β-defensins (hBDs) in mucosa has led to recognition that they are integral in innate immune protection; shielding mucosal surfaces from microbial challenges. The three hBDs, hBD1–3, are cationic, beta-sheeted peptides varying in length from 33 to 47 amino acid residues that are primarily expressed by epithelial cells (1–4). Of the three defensins, hBD-3 is the most positively charged (+11), followed by hBD-2 (+6) and hBD-1 (+4) (4). In solution, hBD-1 and hBD-2 are monomers while hBD-3 exists as a dimer (4). All three proteins have broad-spectrum antimicrobial activity, with hBD-3 consistently being the most potent (4). In addition to their antimicrobial activity, the expression of hBDs in numerous tissues has been linked to their ability to cross-talk with the adaptive immune response by acting as chemokines (5–10). These, along with evidence that some also play a role in wound healing (11), have contributed to our understanding that the hBDs, function to protect us at mucosal surfaces. Interestingly, there is growing evidence that, in certain situations, hBDs can be involved in disease progression, primarily in neoplasia. Abiko et al. (12), were the first to demonstrate the differential expression of hBD-1 and−2 mRNAs in oral cancer cell lines and tumor samples. Since then, multiple studies have reported dysregulation of β-defensins in cancers from diverse anatomical locations within the human body; e.g., oral cavity, esophagus, skin, kidney, prostate, thyroid, liver, lung, colon, vulva, and cervix (Table 1). Human β-defensins can be up- or down-regulated depending on the specific cancer type and its anatomical location. What does this observation mean for hBD expression in the context of neoplasia? Do hBDs have a role to play in activating and/or inhibiting tumor progression? Do hBDs act in unison or does each have a separate and possibly divergent role? Are they only bystander molecules that do not affect the tumor to any appreciable degree? The literature currently is rife with contradictory findings that make it difficult to ascertain what role, if any, hBDs play in neoplasia. In this review, we attempt to organize these observations by scrutinizing the current literature. By segregating findings related to hBD dysregulation in different cancers based on the defensin type and the specific anatomical location of the different cancers, as well as delving into the methodologies used in the studies, we begin to unravel the discrepancies that have plagued this area of research over the last 15 years. We discovered that disparate published results were often due to: (1) studies using cell lines vs. human tissue samples; (2) results based on human tissue samples varied depending upon whether the comparisons were done with matched or unmatched control samples; (3) conclusions varied when using mRNA-based vs. protein-based studies and; (4) in protein-based studies varying methods to detect defensins [Western, Immunohistochemstry (IHC) or Immunofluorescence microscopy (IFM)] could contribute to disparate findings. As examples of contradictory results, some studies showed that hBDs can promote cancer cell migration/proliferation (30, 32, 36, 37) while others showed that defensins were inhibitory (15, 38–42). Interestingly, hBDs were reported to behave as tumor suppressors (15, 22, 39) as well as proto-oncogenes (36, 43, 44). While these results may, on-the-surface, appear to be inconsistent, if studies are segregated based on cell lines used, type of cancer being investigated, and the specific hBD studied, a clearer picture unfolds. In this review, we attempt to set the record straight, and, in summary propose a general model for the role and fate of hBDs in carcinogenesis.

**Table 1:** Studies related to differential expression of hBDs (1, 2, and −3) in cancer.

	**Cancer type/Location**	**Fate in cancer**	**Cell lines/Patient**	**mRNA/Protein**	**Method**	**Sample numbers**	**Significant?**	**References**
HBD-1	Oral cavity (OSCC)	**↓**	Cell lines	mRNA	qRT-PCR	16:15	YES	(13)
		**↓**	Patient (UM)	mRNA	qRT-PCR	5:5	ND	(14)
		**↑**	Patient (UM)	Protein	IHC	30:15	YES	(15)
		**↓**	Patient (UM)	Protein	IHC	60:15	YES	(16)
		**↓**	Patient (UM)	mRNA	Gene-array	355:131	YES	(17, 18)
		**↓**	Patient (M)	mRNA	Gene-array	15	YES	(19)
	Skin(BCC)	**↓**	Patient (M+UM)	mRNA	RT-PCR	22:27	YES	(20)
	Skin (SCC)	**↓**	Patient (UM)	Protein	IHC	23:10	YES	(21)
	Renal Cell	**↓**	Patient (M)	Protein	IHC	48	ND	(22)
	Colon	**↓**	Patients (M)	Both	qRT-PCR/IHC	40	YES	(23)
		**↓**	Patients	mRNA	Gene-array	769:200	YES	(24)
	Prostate	**↓**	Patient (M)	Protein	IHC	100	ND	(22)
	Lung	**↑**	Patients (UM)	Serum protein	RIA	56:46	YES	(25)
		**↑**	Patient (M)	mRNA	RT-PCR	20	ND	(26)
	Liver	**↓**	Patient	mRNA	Gene-array	733:656	YES	(27)
HBD-2	Oral cavity (OSCC)	**↓**	Cell lines	mRNA	qRT-PCR	16:15	YES	(13)
		**↓**	Cell lines	mRNA	qRT-PCR	6:1	YES	(28)
		**↓**	Patient (UM)	mRNA	qRT-PCR	5:5	ND	(14)
		**↓**	Patient (UM)	Protein	IHC	60:15	YES	(16)
	Tonsil	**↓**	Patient (UM)	mRNA	RT-PCR	8:8	YES	(29)
	Esophagus (SCC)	**↑**	Patient (M)	Protein	IHC	58	YES	(30)
		**↑**	Cell lines	Protein	Western	4:1	ND	(30)
	Skin (BCC)	**↑**	Patient (M+UM)	mRNA	RT-PCR	22:27	YES	(20)
	Skin (SCC)	**↑**	Patient (UM)	Protein	IHC	23:09	YES	(21)
	Colon	**↓**	Patient (M)	Both	qRT-PCR/IHC	40	YES	(23)
	Cervix (SCC)	**↑**	Patients (M)	mRNA	RT-PCR	5	ND	(31)
		**↑**	Patients(UM)	Protein	IHC	60:10	ND	(32)
	Vulva	**↑**	Patients (M)	mRNA	RT–PCR	5	ND	(31)
	Lung	**↑**	Patients (UM)	Serum protein	RIA	56:46	YES	(25)
		**↑**	Patient (M)	mRNA	PCR	20	ND	(26)
HBD-3	Oral cavity (OSCC)	**–**	Cell lines	mRNA	qRT-PCR	16: 15	ND	(13)
		**↑**	Patient (UM)	mRNA	qRT-PCR	5:5	ND	(14)
		**↓**	Patient (UM)	Protein	IHC	60:15	YES	(16)
		**↑**	Patient (M)	mRNA	qRT-PCR	4	YES	(33)
		**↑**	Patient (M)	Protein	IHC	4	ND	(33)
		**↑**	Patient (M)	mRNA	qRT-PCR	45	YES	(34)
		**↑**	Patient (M)	Protein	IHC	45	ND	(34)
		**↑**	Patient (UM)	Protein	IHC	29:23	ND	(35)
	Cervical (SCC)	**↑**	Patient (UM)	Protein	IHC	37:22	YES	(36)
	Skin (BCC)	**–**	Patient (M+UM)	mRNA	RT-PCR	22:27	ND	(20)
	Colon	**↓**	Patient (M)	Both	qRT-PCR/IHC	40	YES	(23)

*OSCC, Oral Squamous Cell carcinoma; BCC, Basal Cell carcinoma; SCC, Squamous Cell carcinoma ↓, Down regulated in cancer; ↑, upregulated in cancer; ND, Unchanged in cancer; UM, Unmatched samples; i.e. control samples from different subjects. M, Matched samples; control samples from same subjects, Both, both protein and mRNA; IHC, Immunohistochemistry; RIA, Radioimmunoassay; ND, Not determined/described*.

## Dysregulation of Human Beta-Defensins in Cancer:

### Human Beta-Defensin 1

HBD-1, oddly enough was first described in the hemofiltrate of patients with end-stage kidney disease undergoing dialysis, suggesting renal epithelia as the possible source for hBD-1 (1). Indeed, by *in situ* hybridization, Valore et al. (45), showed that hBD-1 mRNA is present in the epithelial layers of the distal tubules of the loops of Henle, the collecting ducts of the kidney. HBD-1 was also shown to be expressed in human airway epithelia, gingival tissue, epithelia lining the female genitalia and respiratory epithelia of normal and cystic fibrosis (CF) lungs (45–48).

Since its discovery, research has primarily focused on antimicrobial properties of hBD-1 (49–51), although other functions have also been reported (51). These include, immunoregulation (52, 53), cellular differentiation (54, 55), and glucose metabolism (56). HBD-1 has also been shown to be differentially expressed in different types of cancers (Table 1) and has been proposed as a tumor suppressor, because it promotes cancer cell apoptosis (38, 39) and also inhibits migration and invasion of cancer cells (15).

In a large-scale gene expression profiling study of renal epithelial neoplasms, hBD-1 transcript was found to be significantly down-regulated in renal carcinoma (57). IHC staining of the hBD-1 peptide in clinical specimens of both renal cell carcinoma and prostate cancer tissue revealed that >80% of the samples were either completely devoid of hBD-1 expression, or showed marginal expression when compared to adjacent benign epithelium (22). Expression of hBD-1 mRNA and peptide were significantly reduced in basal cell carcinoma (BCC) of the skin (20) and in cutaneous squamous cell carcinoma (SCC), as compared to healthy skin and precursor lesions (21), and in colon cancer (23). Using publicly available gene-expression data sets of colorectal cancer patients from four different countries, Bonamy et al. (24) further corroborated that the *DEFB1* gene was consistently and significantly downregulated in colon cancer compared to non-tumor colon specimens. Gene expression datasets also suggested that *DEFB1* was significantly down-regulated in liver cancer (27).

On the other hand, hBD-1 peptide and mRNA were shown to be upregulated in surgically resected human lung squamous cell carcinoma (SCC) and adenocarcinoma (AC), when compared to normal tissue samples (26), and hBD-1 serum levels were found to be elevated in lung cancer patients (*N* = 56) when compared to healthy subjects (*N* = 46) (25). Therefore, based on the works cited, the production of hBD-1, in the context of cancer, appears to be cancer type and location dependent. This however, does not appear to be the case with oral cancer (oral squamous cell carcinoma; OSCC), as most of the reported studies showed a decrease in hBD-1 expression levels, while only one reported an increase (Table 1). In a study of five OSCC tumors, hBD-1 mRNA was significantly reduced when compared to healthy gingival tissue (14), and this was corroborated in OSCC cell lines by Joly et al. (13). IHC staining, followed by scoring by two independent pathologists, showed that OSCC tissue (*N* = 60) harbored reduced hBD-1 peptide expression when compared to tissue from 15 healthy subjects (16). Interestingly, Han et al. (15), using the same IHC technique, followed by scoring by three independent pathologists, reported just the opposite results; i.e., hBD-1 was induced in OSCC tissue biopsies (*N* = 30) compared to healthy control biopsies (*N* = 15). The authors conceded that they were unable to detect hBD-1 expression in all of the 15 normal subjects. This is quite unusual, as hBD-1 mRNA and peptide are known to be ubiquitously expressed in mucosal tissues (47, 58, 59). Dale et al. (58) and Kawsar et al. (60) using IHC and IFM, respectively, demonstrated that hBD-1 is expressed in the stratum granulosum and spinosum of normal oral epithelium. Moreover, Han et al. (15) showed that hBD-1 expression levels in OSCC were reduced when compared to oral leukoplakia, a dysplastic and premalignant disorder of the oral cavity; i.e., supporting the notion that hBD-1 is under-expressed in OSCC. It has also been shown by merging of multiple microarray datasets of differentially expressed genes (DEGs) that *DEFB1* is down regulated in 355 OSCC tumor samples compared to 131 normal (healthy) controls (17, 18). Therefore, with the exception of the Han et al. (15) findings, the consensus appears to be that hBD-1 is under-expressed in OSCC. It is important to note that hBD-1 expression levels were not compared to matched controls in the studies cited above. Suhr et al. (19) however, compared hBD-1 transcript in OSCC tissue (15 subjects) with adjacent normal tissue and found hBD-1 transcript to be down regulated; further supporting the consensus that hBD-1 is reduced in OSCC.

### Human Beta-Defensin 2

**Figure 1:**
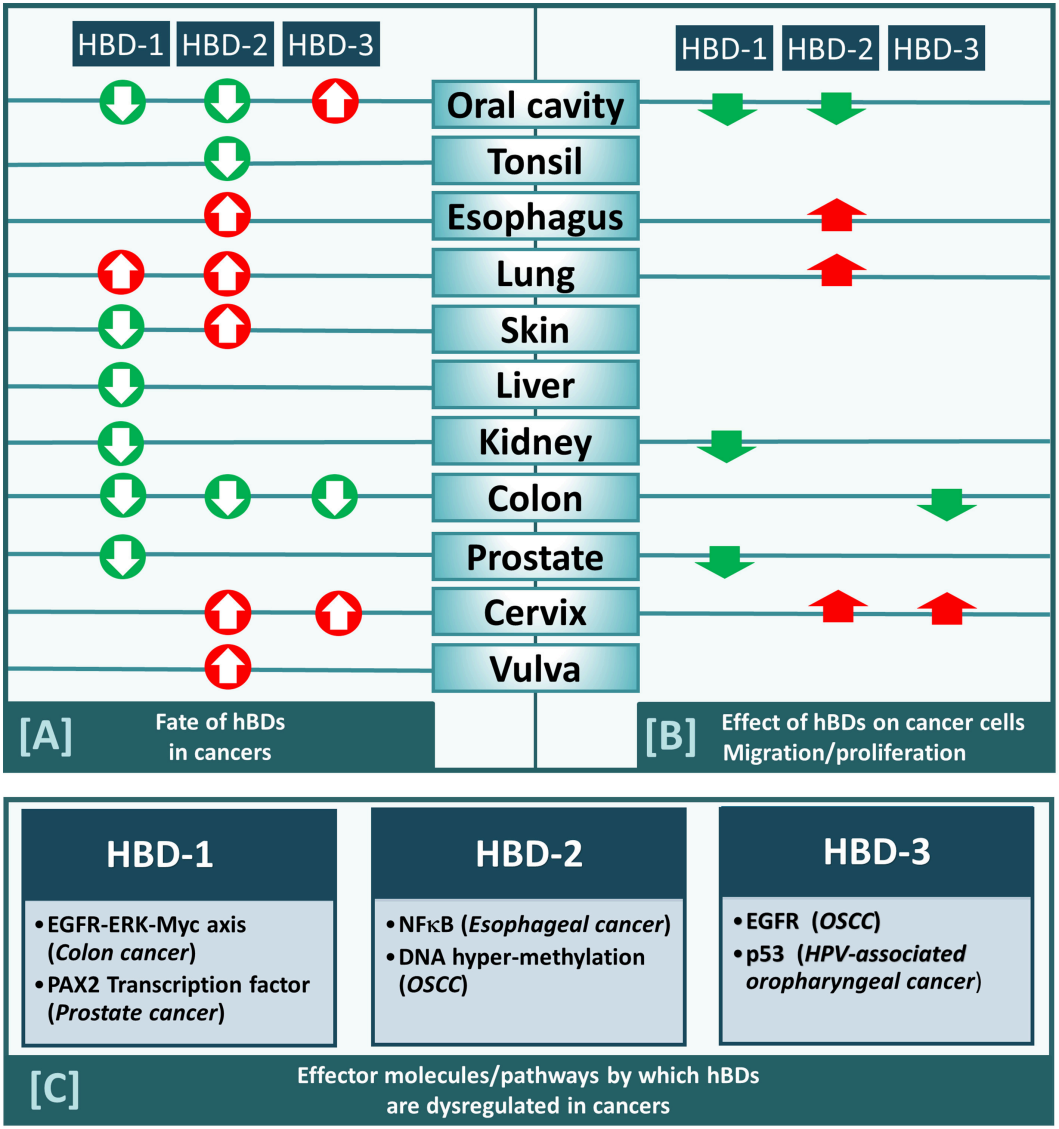
**(A)** HBD-1,-2, and−3 either increased (red arrow) or decreased (green arrow) in cancers from different anatomical locations within human body (as indicated). **(B)** HBD-1,-2, and−3 either promote (red arrow) or inhibit (green arrow) migration/proliferation of cancers cells derived from different anatomical locations (as indicated). **(C)** Effector molecules/pathways by which each of the three defensins (hBD-1,−2, and−3) are dysregulated in cancers.

HBD-2 was discovered in human skin in 1997 (3), followed by descriptions of its expression throughout the epithelia of many organs, including the lung (61). Like hBD-1, the expression level of hBD-2 in cancer tissue appears to be dependent upon the type of cancer studied. While hBD-2 has been reported to be upregulated in several cancers, including esophageal (30), lung (25, 26), cervical (32), and skin cancer (BCC and SCC) (20, 21), it has been reported to be downregulated in colon cancer (23) (Table 1; Figure 1A).

In OSCC tissue, several investigators have reported hBD-2 (both mRNA and peptide) to be downregulated (Table 1) and its' mRNA is reduced in OSCC cell lines compared to normal oral cell lines (13). Its' mRNA was also shown to be reduced in tonsillar cancer (29). Consensus, therefore, supports the observation that hBD-2 is reduced in orally related cancers.

Lisovskiy et al. (31) reported that expression of hBD-2 mRNA is found in tumor cells of the cervix and vulva, but not in adjacent normal cervical and vulvar cells. HBD-2 mRNA absence from these normal cells is contradicted by Markeeva et al. (32) who reported that IHC analysis revealed hBD-2 expression in the adjacent normal cervical epithelium surrounding tumors. Interestingly, although hBD-2 was reported to be expressed primarily in the basal layer of normal cervical epithelial tissue, in carcinoma *in situ* (CIS), hBD-2 is expressed throughout all the tissue layers (32). These results are in stark contrast to immunofluorescence staining results by Jin et al. (44), showing that, in normal oral epithelium, hBD-2 was expressed mainly in the differentiated spinosum and granulosum layers (60), with markedly reduced expression in CIS.

### Human Beta-Defensin 3

HBD-3 was first isolated from human psoriatic scales (2), but was also found in other epithelial tissues, including, tonsils, esophagus, trachea, colon and cervix (2, 23, 36, 62, 63). HBD-3 is particularly interesting in the context of mucosal dysplasia such as CIS and OSCC. It is primarily expressed in the basal keratinocyte region of the oral mucosa; i.e., in highly proliferating epithelial cells (60), has a role in normal wound healing (11), and is induced through activation of epidermal growth factor receptor (EGFR) (60), a process amplified in OSCC (34). Over the last decade, a number of investigators have interrogated the levels of hBD-3 expression in various stages of OSCC. An IHC study demonstrated higher levels of hBD-3 expression in OSCC compared to healthy and dysplastic oral tissues (35). Transcriptional studies demonstrated that hBD-3 mRNA expression is higher in OSCC lesions compared to oral tissue from healthy subjects (14). IHC analysis also showed that hBD-3 peptide was more prominent in oral tumor tissue compared to healthy oral mucosa in the same subjects (34), and that elevated expression of hBD-3 peptide localized exclusively to the cytoplasm of malignant epithelial cells (33). Collectively, these results (Table 1) suggest that hBD-3 is frequently overexpressed in OSCC, and this defensin may have a role in oncogenesis. In support of this hypothesis, Dasgupta et al. (43), reported that the oncogene E6 of HPV 16, etiologically associated with oropharyngeal cancers (64), increases hBD-3 mRNA and peptide expression in infected oral epithelial cells and that p53, an important tumor suppressor inhibited by E6 (65), blocks hBD-3 expression.

As mentioned earlier, Joly et al. (13), by comparing β-defensin mRNA levels in oral epithelial cell lines from 16 OSCC and 15 healthy subjects, found reduced levels of hBD-1 and−2 mRNA in OSCC cell lines compared to normal primary epithelial cells; however, they saw no differences in hBD-3 mRNA levels between the two cell types. While this may appear to contradict the patient-based results (14, 33–35), the Joly et al. (13) study was based on cell lines whose gene expression profiles may not always reflect a true representation of the disease process. As demonstrated by Ertel et al. (66) the signaling and metabolic pathways in transformed cell lines have distinctly different gene expression patterns than those associated with healthy and tumor tissue. In cervical cancer, similar contradictory results were reported in cell lines vs. tumor tissue samples; i.e., strong hBD-3 staining was observed in the cervical cancer tissues, while the peptide was not detectable in three cervical cancer cell lines (HeLa, CaSki, and SiHa) (36). Moreover, we find it perplexing that even though Joly et al. (13) reported no change in hBD-3 transcript expression between OSCC and normal cell lines, this is not reflected in the title of their study “*Loss of human beta-defensin 1, 2, and 3 expression in oral squamous cell carcinoma*.” Another perplexing result comes from an *in situ* hybridization study by Yoshimoto et al. (67). They reported that out of 20 OSCC samples, only 4 were positive for hBD-3. We find that quite unusual. While hBD-3 peptide may have varied between samples, all should have been hBD-3 transcript positive, suggesting that the *in situ* hybridization may not have been optimal in all the cases.

In an IHC study by Wang et al. (16) hBD-3 was shown to be under-expressed in OSCC when compared to healthy oral tissue. Examination of the details of this work reveals that: (1) the sample cohort was over-represented by OSCC subjects (60 OSCC vs. 15 healthy); (2) all 15 “healthy” samples were collected from surgical extractions of impacted third molars, a specific location of the oral cavity that may not be representative of the various sites from which the OSCC samples were taken and; (3) it is clear from (2) that the authors did not use paired samples to overcome inherent geographic differences in the oral cavity. On the other hand, observations reported for hBD-3 mRNA and peptide levels by Kestling et al. (34), and Shuyi et al. (33), were obtained from matched paired samples from the same subjects. Additionally, Jin et al. (44) demonstrated, by quantitative IFM, that hBD-3 is over-expressed in CIS compared to healthy tissue; hence, it is unlikely that hBD-3 would be overexpressed in CIS but not in OSCC. Finally, while unrelated to hBD-3 directly, it bears mentioning that Wang et al. (16), also reported that NOD1 and RIP2 were under-expressed in OSCC compared to healthy oral tissues; findings that are contradictory to more recent results showing that both proteins are over-expressed in OSCC (68). In summary, the majority of studies agree on hBD-3 overexpression in OSCC compared to healthy paired control tissue. Interestingly, hBD-3 overexpression has also been reported in cervical cancer (36) but not in colon cancer (23). Therefore, as we concluded for hBD-1 and−2, dysregulation of hBD-3 in cancer tissue appears to be associated with the type of cancer being studied and its location.

## Mechanisms of Beta-Defensin Dyregulation in Cancers

The pathway by which a particular β-defensin is dysregulated in cancers has been studied in only a few cancer types and has been found to vary depending on the hBD and the cancer it is associated with (Figure 1C). For example, hBD-1 has been shown to be suppressed in colon cancer through the EGFR-ERK-MYC axis (24), while in prostate cancer it is suppressed by the PAX2 transcription factor (69). Mechanisms for dysregulation of hBD-2 and−3 are also cancer type dependent. For hBD-2, the nuclear factor kappa b (NF-κB) pathway was found to regulate it in esophageal cancer (30), while in OSCC it is regulated by DNA hypermethylation (28). For hBD-3 the EGFR pathway activates it in OSCC (33), while in HPV-associated oropharyngeal cancer it is regulated via the tumor suppressor p53 (43).

## The Effect of Human Beta-Defensins on Cancer Cell Migration/Proliferation

Addition of exogenous synthetic hBD-1 peptide inhibits bladder cancer cell (TSU-Pr1) proliferation (38). Overexpression of hBD-1, by stable transfection of *DEFB1* cDNA in renal cancer cells (SW156) resulted in caspase-3-mediated apoptosis (38). Induction of hBD-1 by ectopic expression of *DEFB1* cDNA in prostate cancer cell lines PC3 and DU145 (39) also resulted in decreased cell growth and viability. HBD-1 also caused rapid induction of cytolysis and caspase-mediated apoptosis in these cell lines (39). Similarly, hBD-1 overexpression by stable transfection of *DEFB1* has also been shown to inhibit migration of several oral cancer cell lines (HSC-3, UM1, SCC-9 and SCC-25) (15). RhoA and RhoC are likely to be associated with the regulatory effect of hBD-1 on the migration and invasion of OSCC cells (15). Thus, hBD-1 appears to have anti-cancer and/or tumor suppressive activities, and therefore its suppression in prostate, renal, bladder and/or oral cancer might contribute to cancer cell survival and tumor progression. Interestingly, hBD-1 is under-expressed in all these cancer types (e.g., renal, prostate and oral; Table 1; Figure 1A).

Increased expression of hBD-2 generated by gene transfection inhibits the proliferation and invasion of the OSCC cell line (SAS), possibly *via* G1/S arrest and pRB gene expression, indicating that hBD-2 may act as a tumor suppressor (28). Interestingly, as described above, hBD-2 has been reported to be under-expressed in OSCC (Table 1; Figure 1A). In contrast, it is overexpressed in esophageal, lung, and cervical cancer and it appears to promote the growth of cell lines from these cancers; i.e., esophageal (KYSE-150 cells, through NF-κB) (30), lung (A549 cells, through ATP-binding cassette transporter G2) (37), and cervical (M-HeLa) cancer cell lines (32). Thus, in cancers where hBD-2 under-expression has been reported, it inhibits migration and proliferation of cell lines emanating from those cancers, while in cancers where it is overexpressed it promotes respective cancer cell growth.

Over-expression of hBD-3 in HeLa cells (cervical cancer cell line), using lentiviral constructs, promoted cell proliferation by accelerating G1/S progression and enhanced cell migration and invasion (36), while addition of exogenous hBD-3 in colon cancer cell lines (SW480, SW620) inhibits their migration via downregulation of MTA2 (42). *In vitro* observations have also reported that exogenous hBD-3 promotes directed chemotaxis of myeloid cells via the chemotaxis receptor CCR2 (44). Moreover, *in vivo* observations show that in CCL2 (chemokine ligand for CCR2) deficient oral CIS lesions, hBD-3 over-expression acts to selectively recruit tumor-associated macrophages to CIS lesions (44). These results, collectively, suggest that in those cancers where hBD-3 over-expression has been reported, principally in oral, and cervical cancers, hBD-3 acts to exacerbate neoplasia, while in cancers where it is under-expressed, principally colon cancer, it is associated with inhibition of neoplasia.

## Take Home Message

Based on the literature available, the apparent contradictory findings between labs regarding β-defensin expression levels in cancer tissue appear to be due to levels reported in specific cancer types (Figure 1A), where a given defensin is increased in one cancer but decreased in another. The mechanism by which hBDs are dysregulated in cancer also varies depending on which defensin and cancer type are being studied. Additionally, a specific beta defensin may promote or inhibit cancer cell proliferation/migration depending on the origin of the cancer cell, and the outcome may be associated with whether the defensin is increased or decreased in the tumor from which the cells are harvested (Figure 1B). Therefore, generalizations as to the role of β-defensins in cancer and neoplasia should be avoided, as the function of defensins may differ between cancers and cancer cells.

## Concluding Remarks

We have surveyed the literature in an attempt to identify potential reasons behind contradictory findings related to differential regulation of β-defensins in different cancers. By mining the published information, interrogating methodologies and scrutinizing the subject cohorts used in the studies, we have endeavored to investigate the inconsistencies related to the fate of β-defensins in OSSC, and have concluded that hBD-1 and−2 are down while hBD-3 is upregulated in OSCC. Additionally, after evaluating each study based on the specific defensin and the anatomical location of the respective cancer, we conclude that generalizations as to the fate of the β-defensins in cancer should be avoided; they are defensin and cancer specific. We have previously stressed the promiscuous nature of human β-defensins (10), and are therefore not surprised that various cancers demonstrated differential expression of these molecules. These molecules are regulated differently (60, 70–72), they interact with receptors on cells differently and, while some function primarily as AMPs, others, in addition to antimicrobial activity, function in wound healing (11). Defining the role(s) AMPs play in cancer may lead to innovative ways to mine them as potential biomarkers for diagnostic/prognostic purposes and/or in novel therapeutic modalities.

## Author Contributions

SG performed the literature search and drafted the manuscript. AW contributed to the discussions and writing of the article. TM provided critical revisions.

### Conflict of Interest Statement

The authors declare that the research was conducted in the absence of any commercial or financial relationships that could be construed as a potential conflict of interest.
